# Assessment of the Morphology and Degenerative Changes in the Temporomandibular Joint Using CBCT according to the Orthodontic Approach: A Scoping Review

**DOI:** 10.1155/2022/6863014

**Published:** 2022-02-01

**Authors:** Sebastian Dygas, Izabela Szarmach, Ilona Radej

**Affiliations:** Department of Orthodontics, Medical University of Bialystok, Bialystok, Poland

## Abstract

**Background:**

Available knowledge about disorders of temporomandibular joint structures and their association with orthodontic variables are still lacking.

**Objectives:**

This article is aimed at to identifying studies and presenting current information on the relationship between morphology diversity and the occurrence of degenerative changes in structures of the temporomandibular joint (TMJ) assessed by cone-beam computed tomography (CBCT) in the context of craniofacial morphology and malocclusion. *Search Methods*. The review was conducted by analyzing the PubMed (including Medline), Cochrane Library, Web of Science, and Scopus electronic databases up to November 2021 using two different comprehensive search strategies based on keywords as well as additional manual searches. *Eligibility Criteria*. Selection of the literature was carried out according to the PRISMA-ScR checklist. Methodological quality of the selected studies was evaluated using JBI Critical Appraisal Tool.

**Results:**

The electronic databases search revealed 3331 records. After applying the eligibility criteria and JBI assessment, a total of 33 studies were extracted and selected to the study. The review was divided into 4 parts, in which the following correlations were assessed in terms of orthodontic variables: TMJ degenerative changes, joint space and condylar position, condylar shape, TMJ articular eminence, and fossa.

**Conclusions:**

Skeletal and dental class II malocclusion with a retrognathic mandible, a hypodivergent skeletal pattern with a steep mandibular plane, and significant lateral mandibular displacement can be risk factors for developing radiographically detectable degenerative changes. Patients with skeletal and dental class III malocclusion as well as a hyperdivergent skeletal pattern may be at greater risk of TMD occurrence compared with other groups. Further studies are necessary to clarify the relationship between the position of the condylar processes and the presence of degenerative changes in the temporomandibular joints among orthodontic patients.

## 1. Introduction

Cone-beam computed tomography (CBCT) in dentistry was implemented more widely in Europe in 1998 [1] and in the USA in 2001 [2]. Currently it is being used, among other things, for imaging of the bony structures of the temporomandibular joint (TMJ) and has also emerged as the procedure of choice for the detection and evaluation of TMJ degenerative lesions [3–5]. CBCT of the temporomandibular joint is also used for individual measurements of the joint space and the position of the condylar process and its volume, as well as the articular eminence (height and inclination) [6]. Assessment of the articular disc is performed using MRI [7].

According to recommendations, CBCT is only performed in cases where it will significantly influence treatment decisions. This is the case, for example, in advanced degenerative joint diseases (DJDs) or in severe, symptomatic TMD [8]. Diagnosis and classification of degenerative lesions and pathologies as well as detailed assessment of TMJ structures should be undertaken according to RDC/TMD criteria [9]. This is usually a qualitative assessment performed by an experienced clinician. Quantitative assessments of the degree of inclination of the articular eminence, glenoid fossa depth or joint space measurements due to ethnic differences, and the location of additional structures (joint discs), as well as a lack of established standards for the range of values obtained, have proven to be problematic. Researchers such as Ikeda and Kawamura have attempted to tackle these issues by assessing the optimal position of the articular heads in the sagittal section at three points (anterior, superior, and posterior joint spaces) in patients without signs of dysfunction and showing compliance of maximal occlusion with the central relation based on MRI and CBCT [10]. In the case of joint space, they proposed to use the measurement methodology and results of their own research as a starting point for assessing the correctness of the position of the condylar processes in the joint.

The relationship between the symptom severity of TMJ dysfunction and the occurrence of degenerative lesions has been long debated among orthodontists. Some studies indicate that the presence of radiographically detectable remodeling is associated with a clinical diagnosis of temporomandibular disorders (TMDs), Helkimo index, malocclusion, and age and gender [11–14]. In addition, the shape of the mandibular condyle and its position in the glenoid fossa may also influence the occurrence of degenerative changes in the TMJ [15, 16]. However, some researchers claim that there is no relationship between the severity of the lesions seen on CBCT and clinical symptoms [17–19].

The prevalence of degenerative lesions in the joint differs according to various authors and studied populations. In a Korean population, the most commonly observed are the following, in descending order: sclerosis (30.2%), erosions (29.3%), flattening (25.5%), and deviations in form (13.2%) followed by osteophytes (8.0%) and subcortical cysts (5.5%) [16]. Kiliç et al. [18], on the other hand, noted in a Turkish population with diagnosed osteoarthritis (OA): erosions, flattening, osteophytes, and sclerosis, with subchondral cysts being the least common. Rehan et al. also reported differing results [19] in a paper that compared Egyptian patients diagnosed with OA with a control population. They found the studied group had (also in descending order) flattening, erosions, sclerosis, and also osteophytes and subchondral cysts.

The position and morphology of the TMJ are determined by a number of factors such as gender, age, growth pattern, functional disorders, or intra-articular pathologies. Abnormalities in muscle tone or disorders in occlusal relations have been reported to cause uneven force distribution and remodeling of joint surfaces resulting in consecutive adaptation to individually changing mechanical and functional conditions in the joints and surrounding structures [9, 20]. With adaptive remodeling in the TMJ, the anatomical position of the condyle changes. Thus, some researchers believe that malocclusion and craniofacial morphology may play a key role in the position of the joint heads [21, 22]. Other authors argue that there is no relationship [23, 24]. A different view suggests that variation in the functional loading of the condyle during the act of mastication is determined by craniofacial structure and may impact diversity in joint morphology [25]. There is also a lack of agreement in assessing the effect of centric occlusion-centric relation (CO-CR) discrepancy on the position of the condyles in the glenoid fossa [26, 27].

The shape of the condyle, according to some researchers, varies depending on the type of malocclusion [28, 29]. The relationship between occlusal forces and condylar morphology in different skeletal configurations may be important and affect the stability of orthodontic treatment outcomes [20, 30, 31].

An integral part of the TMJ complex is the articular eminence and the glenoid fossa. They are characterized by the depth of the fossa and the height and angle of the eminence (“angle of inclination” or “steepness of the articular eminence”), which significantly affect the range and inclination of the articular pathway as well as the degree of rotation of the articular disc [32]. A relationship has been shown between the fossa-eminence morphology and temporomandibular joint disorders and internal derangements [33]. This variability may influence the incidence of TMD or radiographically detectable changes in orthodontic patients [28, 34, 35]. However, according to other researchers, craniofacial architecture, occlusion, and articular eminence inclination are not associated with an increased prevalence of temporomandibular joint dysfunction [36].

It seems that there is a lack of summary of studies concerning which malocclusions and which cephalometric variables may be connected with an increased risk of developing degenerative changes in the TMJ. For this reason, it is challenging to assess which types of skeletal and dental discrepancies should be routinely investigated in the clinical setting along with a CBCT of the joint.

This study is aimed at reviewing the literature including studies on the anatomy, as well as the disorders in the structures of the temporomandibular joint in the context of maxillofacial morphology and malocclusion, and outlining the current knowledge in this field.

## 2. Materials and Methods

### 2.1. Protocol and Registration

This scoping review was based on the Preferred Reporting Items for Systematic Reviews and Meta-Analysis extension for Scoping Review (PRISMA-ScR) statement protocol. The population, intervention, comparison, and outcome (PICO) approach was defined as follows: population: pediatric and adult orthodontic patients; intervention: CBCT of the TMJ; comparison: between patients with the presence and absence of TMJ degenerative changes, between patients with different condylar positions and joint spaces, between patients with different condylar shapes, and between patients with different TMJ articular eminences and fossae morphologies; outcome: relationship between craniofacial morphology and the differences in TMJ anatomy as well as the occurrence of bony degenerative changes; and time period: published between 2009 and 2021.

### 2.2. Data Sources and Search Strategy

Four databases were analyzed over the time range given below:

- PubMed (including MEDLINE) (database root [1966]-November 01, 2021)
- Web of Science (database root [1965]-November 01, 2021)
- Cochrane Library (database root [1991]-November 01, 2021)
- Scopus (database root [1965]-November 01, 2021)

Article searches were conducted using free text terms and controlled vocabulary (MESH) shown in the two schemes below:

The first scheme is as follows:

1. (“cone beam computed tomography” OR “CBCT”)

AND

(2) (“temporomandibular joint” OR “TMJ”) OR (“TMD” OR “temporomandibular disorder”)

AND

(3) free text terms and controlled vocabulary (MESH) with the highest number of records referring to orthodontics (especially malocclusion)

The second scheme is as follows:

1. (“temporomandibular joint” OR “TMJ”) OR (“TMD” OR “temporomandibular disorder”)

AND

(2) free text terms and controlled vocabulary (MESH) with the highest number of records referring to orthodontics (especially malocclusion)

AND

(3) free text terms and controlled vocabulary (MESH) with the highest number of records referring to the anatomy and pathology of the temporomandibular joint

In addition, a manual check of references in systematic reviews on similar topics and in selected original papers was performed to find potentially useful articles.

The specific headwords, along with the number of records, used in Sections 2.2 and 2.3 are presented in the Supplementary Materials (appendix) (available here).

### 2.3. Eligibility Criteria

The inclusion and inclusion and exclusion criteria are outlined in Table 1.

### 2.4. Search Results

**Figure 1:**
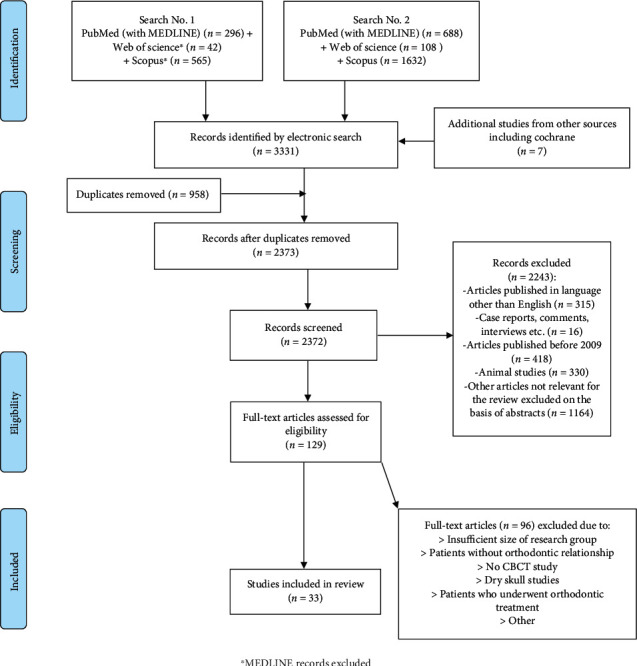
Preferred Reporting Items for Systematic Reviews and Meta-Analysis (PRISMA) search strategy flowchart; ^∗^MEDLINE records excluded.

The initial search, conducted according to the above rules, yielded 3331 records, which were entered into the EndNote X9 software (Clarivate Analytics, Philadelphia, United States) to filter out duplicates. In the next stage, after both automatic and manual removal of duplicate articles, 2373 records were obtained. Applying inclusion and exclusion criteria and an analysis of abstracts resulted in the inclusion of 129 papers for further systematic review. Finally, after analysis of the full texts by two independent researchers, 33 original papers remained as the basis for this article.  Figure 1 shows a detailed flowchart of the systematic review search and selection process according to the PRISMA statement.

### 2.5. Critical Appraisal of Individual Sources of Evidence

Two of the 33 original papers included in the review were described by the authors as cross-sectional studies and the remainder as retrospective comparative and/or observational studies. The Joanna Briggs Institute (JBI) Critical Appraisal Checklist For Studies Reporting Prevalence Data was used to assess the quality of the included studies [37]. The examination of research evidence was conducted by two independent investigators according to a form (a list of 9 questions), assessing the study design, conduct, and reliability of the results (yes, no, unclear, and not applicable). Disagreements between researchers were discussed in order to come to a consensus. In particular, attention was paid to the following: study group selection and allocation, research methods, and statistical analysis.

### 2.6. Data Analysis

All results were analyzed descriptively.

## 3. Results

### 3.1. CBCT Assessed Degenerative Changes in the Temporomandibular Joint Related to Orthodontic Patients

Of the 33 papers included in the review, only 6 articles addressed the association of degenerative changes in the TMJ, diagnosed by CBCT, with maxillofacial morphology and malocclusion [11, 38–42]. These studies were published between 2012 and 2021 and included patient populations from various countries: Brazil [40], China [38], Latvia [11], Taiwan [41], South Korea, [39], and the USA [42]. Sample sizes ranged from 83 [38] to 273 [40] patients. A summary of the descriptive characteristics of included articles is provided in Table 2.

#### 3.1.1. CBCT Assessed Degenerative Changes in the Temporomandibular Joint in Different Sagittal Skeletal Patterns

Walewski et al. [40] on a group of patients diversified by malocclusion established that nearly 6% of condyles presented more than one type of degeneration at the same time, and a total of 52.3% had radiologically detectable changes within the hard tissues, of which flattening was the most common. Nearly one-third of the articular eminences showed the presence of degenerative changes in the osseous structure. In skeletal classes I and III, condylar flattening was the predominant degeneration within the condyle, while in grade II, osteophytes and flattening were detected equally prevalent. The researchers found no significant difference in the occurrence of degenerative TMJ changes among groups that included different skeletal classes, age, or gender. No association was also detected between patients divided into groups according to the RDC/TMD classification.

Chen et al. [38], on the other hand, estimated that among the patients included in their study (skeletal class II malocclusion), more than half showed changes in the hard tissues of the TMJ, of which 1/3 were characterized by bilateral osteoarthrosis. The most common degenerative lesions were condylar shortening (18.6%), osteophytes (15.3%), surface erosions (14.2%), and generalized sclerosis (7.1%). Subcortical cysts were not observed. SNB and ANB angles presented a statistically significant association with degenerative changes.

Krisjane et al. [11] differentiated their studied population according to skeletal classes and noted that almost half of the TMJs were characterized by radiographically detectable lesions within the hard tissues, of which surface flattening (39.8%), osteophytes (21.0%), condylar hypoplasia (15.5%), and erosions (9.5%) were observed most frequently. Only a few flattening and subcortical sclerosis lesions were observed within the articular eminences in the entire study group. Patients of the class II skeletal group showed the statistically highest incidence of degenerative changes within the TMJ. The control group (skeletal class I) marginally demonstrated a presence of remodeling or degenerative changes in the joint in contrast to skeletal class III (almost half of the patients) and skeletal class II (more than 2/3 of the patients). Based on these results, the authors suggested that severe skeletal defects were associated with a relatively high risk of dysfunction and remodeling of temporomandibular joint structures.

Tran Duy et al. [41] in a homogeneous study group (women with skeletal class III defect with chin deviation) evaluated the presence of degenerative changes in the TMJ by CBCT independently in three projections according to an individually prepared scale scored from 0 to 20. The volume of the condyle was also investigated, and a correlation of the results with chin deviation was assessed. It was found that the presence of degenerative changes (in this case, more than 10 points on the scale) was statistically associated with severe chin deviation (≥3 mm). In addition, a difference in mandibular condyles volume in the same patient and subcortical sclerosis in 1 or 2 joints were also associated with severe chin deviation.

#### 3.1.2. CBCT Assessed Degenerative Changes in the Temporomandibular Joint in Different Vertical Facial Patterns

Kang et al. [39] studied a group of age-matched patients divided via the RDC/TMD classification according to the presence of dysfunction and degenerative changes in the TMJ. The group of patients without signs of TMD but with diagnosed OA displayed a significantly more retrognathic mandible, a markedly increased FMA angle, a greater *y*-axis to SN and SN-GoMe angle, and a lower facial height ratio (S-Go/N-Me). Clinically, this translated into a distinctly hypodivergent profile and a steeper mandibular plane compared to the control group and the TMD group without OA. The differences reported were statistically significant. The researchers also concluded that TMJ OA may be associated with a considerable delay in dental development; therefore, clinicians should pay more attention to this aspect when evaluating adolescent patients for degenerative changes.

Dadgar-Yeganeh et al. [42], on a group of patients diversified by the presence of DJD, established that ramus, mandibular, and condylar height were substantially smaller when compared with a control group. They also noticed that occurrence of DJD is correlated with long vertical facial dimensions, as well as a significantly narrower cross-sectional area of the upper airway. The reported differences were statistically relevant. It was found that long facial types could be associated with degenerative TMJ changes and condylar growth disruptions.

Chen et al. [38] noted that of the three groups classified at baseline, the following cephalometric variables presented a statistically significant association with degenerative changes: mandibular plane angle (MP-SN), Pg to *y*-axis distance, posterior facial height (S-Go), and facial height ratio (S-Go/N-Me). Their study showed that patients with a class II skeletal malocclusion and concomitant degenerative changes in the TMJ had reduced posterior facial height (S-Go), enlarged mandibular plane angle (MP-SN), and the most retrusive mandibular morphology.

#### 3.1.3. Results of Individual Studies: Summary

Comparing the results of the above papers, most authors associate the occurrence of degenerative changes with severe class II malocclusion [11, 38, 39], major maxillofacial defects including class III malocclusion [11, 41], or a hypodivergent profile with high mandibular plane angle [38, 42]. Due to the high variability of the study groups in the articles evaluated in this subsection, it is not possible to directly compare the findings with each other.

### 3.2. TMJ Condylar Shape in CBCT Related to Orthodontic Patients

Variability of mandibular condylar head surface morphology in correlation with maxillofacial morphology and malocclusion was evaluated in 5 papers included in a systematic review [20, 24, 43–45]. These studies were published between 2009 and 2019 and included patient populations from: Turkey [45], Brazil [44], Japan [20], South Korea, [24], and the United States [43]. Sample sizes ranged from 40 [20] to 910 [45] patients. A summary of the descriptive characteristics of the included articles is provided in Table 3.

#### 3.2.1. TMJ Condylar Shape in CBCT in Different Sagittal Skeletal Patterns and Angle's Classification

Yalcin and Ararat [45] evaluated the shape of the condyles in the coronal projection in a group of patients and divided them according to Angle's class molar relationship into classes I, II, and III. The most common types observed were as follows: convex (40.6%), angled (34.3%), flat (15.4%), and round (9.7%). Statistically significant correlation was detected only on the right side. The authors noted that the majority of patients with skeletal class II were characterized by a convex shape of the condyle.

Merigue et al. [44] studied the condylar processes, similar to Yalcin and Ararat, in a coronal projection in groups of patients showing class I and II division 1 molar relationship. The shapes of the articular heads were divided into 3 groups: flat or convex, round, and triangular or angled. The most common condyle shape in both groups was a convex shape (57.7% for the control group and 75.1% for the study group, respectively). The authors found no statistically significant differences between the studied groups.

Kurusu et al. [20] evaluated the mutual correlation of malocclusion, joint head shape, and joint loading. Measurements were made in the coronal and axial projections. A positive correlation was found between the mandibular plane value angle (MP-FH) and occlusal forces. In addition, it was found that the condylar processes that received less load were correspondingly smaller in size. No correlation was found between malocclusion and the shape or size of the mandibular condyles.

#### 3.2.2. TMJ Condylar Shape in CBCT in Different Vertical Facial Patterns

Park et al. [24] evaluated the morphology of the articulations in the sagittal projection in a group differentiated by the growth pattern of the bone bases. The total of the shapes detected corresponded to the normal (72.5%), flattened (20%), osteophytic (7.5%), and unclassified (0%) groups. The authors showed that patients in the hypodivergent group had larger condylar processes with a predominantly oval shape, while smaller and rounder articular heads were more common in the hyperdivergent group.

Contro et al. [43] in a large group of patients characterized by growth pattern of bone bases and mandibular symphysis evaluated the condylar processes in CBCT using Procrustes analysis for the shape evaluation. The study showed that in brachyfacial patients, the processes were more convex in the anterior surface and more concave in the same area in dolichofacial patients. Contrary to the generally held view, brachyfacial patients had similar mean values to the obtuse chin angle group, while dolichofacial patients had similar variables to the acute chin angle group.

#### 3.2.3. Results of Individual Studies: Summary

In conclusion, there is no complete consensus on the correlation of condylar shape with malocclusion. Most researchers agree that a convex or flat shape is usually found in the general population. Some studies show that convex shape of the condyles is most common in class II malocclusions, similar to brachyfacial/hyperdivergent patients. The dolichofacial/hypodivergent group is more likely to have a gentler, less steep shape of the joint heads.

### 3.3. Joint Space and Condylar Position in TMJ in CBCT Related to Orthodontic Patients

The association of joint space features and condylar positions with maxillofacial morphology and malocclusion was addressed in 19 articles included in the systematic review. These studies were published between 2012 and 2021 and involved patient populations from China [22, 46], Brazil [35, 44, 47], India [48, 49], Peru [34], Iran [25], Nepal [50], Turkey [51, 52], South Korea [24, 53–55], Egypt [56, 57], and Ukraine [58]. Sample sizes ranged from 20 [50] to 180 [35] patients. A summary of the descriptive characteristics of included articles is provided in Table 4.

#### 3.3.1. Results of Individual Studies

In most papers, homogeneity was maintained in the selection of the age distribution of the studied group and only adults or only children were included. In four of the collected articles, the study group was mixed [22, 44, 46, 58]. In all publications, with one exception [22], both women and men were studied. Only 4 papers were linear measurements performed by more than 1 researcher [34, 35, 56, 57]. The position of the condyle and spatial analysis was evaluated most often in the sagittal plane.

The mandible of the patients during CBCT examination was positioned according to maximum intercuspation, with the exception of one case where radiological imaging was performed in both central relation and maximum intercuspation [47].

#### 3.3.2. Joint Space and Condylar Position in TMJ in Different Sagittal Skeletal Pattern and Angle's Classification

Papers that evaluated the relationship of dentoskeletal malocclusion in the sagittal plane to the position of the condyles and joint space analysis present with very diverse results.

Class II malocclusions were most often characterized by narrowing of the dimensions of the anterior [25, 35, 46], upper [52], or both anterior and upper [34, 51] joint spaces. This resulted in an anterior, superior, or anterosuperior position of the condylar heads, respectively, as compared with the other groups. Some papers did not find a statistically significant association between altered joint space morphology and condyle position and skeletal or dental class II malocclusion [44, 47, 52, 54, 55]. Only two articles reported a retrusive position of the mandibular condyles, which resulted in a wider dimension of the anterior articular joint space [48, 57].

In skeletal and dental class III malocclusions, the most common deviation was narrowing of the upper [46, 57] or upper and posterior [35] joint space, resulting in an anteroposterior position of the condyles compared with the other groups. One study showed that in a class III malocclusion, as in a class II (both with increased vertical facial dimensions), the condyles were positioned more anteriorly and superiorly compared to the control group [34]. In another study, the authors did not find an optimal condyle position in any of the patients presenting with a class III malocclusion [58]. A significant number of the articles included in the review did not prove a statistically significant correlation between mandibular head position and articular joint space morphology and anterior malocclusions [25, 47, 50, 52, 54, 55].

Only one study, but based on a large study group (120 patients), evaluated the effect of the extent of chin deviation in skeletal and dental class III malocclusion on the position of the condyle in the joint space. The authors observed no difference between the two groups (deviation > 2 mm and >3 mm) for the anterior and posterior joint spaces and a statistically significant difference in the upper joint space—lower values were obtained for the group with greater lateral displacement of the chin. This may have played a role in the increased incidence of TMD in this group. On the basis of the presented results, they found that the mandibular condylar process was located anterosuperiorly in class I malocclusions, posterosuperiorly in class II malocclusions, and more anterosuperiorly in class III than in class I malocclusions [48].

#### 3.3.3. Joint Space and Condylar Position in the TMJ in Different Vertical Facial Patterns

Similarly to the previous subsection, studies evaluating the relationship of the vertical facial pattern to the position of the condylar processes and the joint space showed large variations in the results were obtained.

All of the possible spatial combinations of condyle positioning in the joint had been observed in hypodivergent patients. Both narrowing of the anterior [56], anterior and superior combined, [22, 34], and posterior [49], as well as widening of the upper articular talar space, had been demonstrated [24, 54].

In hyperdivergent patients, narrowing of the posterior [22, 56] and upper [24, 54, 55] joint space was most commonly observed. Also, Alhammadi et al. [56] demonstrated widening of the upper joint space in addition to narrowing of the posterior joint space. Some authors did not observe any statistically significant relationships [49].

### 3.4. TMJ Articular Eminence and Mandibular Fossa in CBCT Related to Orthodontic Patients

Of the papers included in the systematic review, 15 articles investigated the correlation of joint eminence/fossa features, as assessed by CBCT, with maxillofacial morphology and malocclusion. These papers were published between 2013 and 2021 and focused on populations from China [22, 46, 59], Brazil [35, 40], Peru [34], South Korea [24, 54, 55], Egypt [56, 57], Turkey [51], India [49], Italy [60], and Iran [61]. Sample sizes ranged from 28 [51] to 213 [40] patients.  Table 5 summarizes the key information and assessed variables included in the articles.

#### 3.4.1. Results of Individual Studies

The age distribution of the patients included in the study was homogeneous in the majority of published papers. The studied groups consisted of adults and children in two of the collected articles [22, 46]. In all studies, with one exception [22], the studied population consisted of both women and men. In half of the papers, the CBCT study was analyzed with more than 1 investigator [34, 35, 40, 56, 57, 59, 60]. The most commonly evaluated variables were the height and angle of the articular eminence and depth and width of the glenoid fossa.

#### 3.4.2. TMJ Articular Eminence and Mandibular Fossa in Different Sagittal Skeletal Pattern and Angle's Classification

Papers that evaluated the correlation of sagittal skeletal pattern and Angle's classification malocclusion with the assessment of articular eminence and glenoid fossa morphology presented a high variety of results. Most often, a decrease in the angle of the articular eminence was observed compared to the control group in skeletal and dental class II malocclusions [34, 57]. In contrary, Fan et al. [59] presented in their study that class II/2 patients were associated with the highest articular eminence inclination. Gorucu-Coskuner et al. [51] showed a deeper and wider glenoid fossa in class II division 1 malocclusions compared with skeletal class II division 2 groups.

In skeletal and dental class III malocclusion, shortening of the articular eminence [57] or shortening of the articular eminence and reduction of the angle of inclination simultaneously were most commonly observed [34, 35]. The glenoid fossa was usually widened [57] or widened and shallowed [46] compared to the control group. One study showed larger values of the glenoid fossa height compared with skeletal class II [55]. Two other studies stated that the widest glenoid fossa is commonly seen in skeletal class I [59, 61] .

No correlation between malocclusion and articular eminence steepness or glenoid fossa width and depth were found in three papers included in this review [40, 54, 60].

#### 3.4.3. TMJ Articular Eminence and Mandibular Fossa in Different Vertical Facial Patterns

Similar to the previous subsection, the relationship of vertical facial patterns with the position of the condylar processes and the joint space showed great variability in the reviewed studies. A reduced articular eminence inclination was usually observed in hypodivergent patients [22, 56]. The glenoid fossa tended to be shallower [22] and wider [56] than the other groups. Hyperdivergent patients typically had higher values for the height [55, 56] and angle of articular eminence inclinations [22]. The glenoid fossa, according to one study, was correspondingly shallower than in the control group [24]. On the other hand, some researchers stated that the glenoid fossa in skeletal deep bite was deeper and wider compared with other groups [55].

However, other researchers did not find a correlation between the angle of the bases and the height [22] and steepness of the eminence [24] and width [22] and depth of the glenoid fossa [49] or the width and depth of the glenoid fossa simultaneously [54].

## 4. Discussion

Clinicians' understanding of the correlation of malocclusion and maxillofacial morphology with the TMJ complex may be important in the diagnosis and planning of orthodontic treatment, during which the initial position of the condyles is often altered.

The bony parts of the selected temporomandibular joint should intentionally be evaluated using cone beam computed tomography. This allows for precise linear measurements with accurate values and three-dimensional reconstruction of hard tissues [62]. CBCT, performed in the standing position, minimizes the occurrence of diagnostic error in assessing the position of the condyles in the joint due to the more physiological position of the patient's head during the examination [53]. Additionally, when several lesions of the bony components of the TMJ are evaluated, it is more reliable and accurate compared to standard computed tomography and pantomographic radiographs.

### 4.1. CBCT Assessed Degenerative Changes in the Temporomandibular Joint: Summary

Degenerative changes in the radiographic appearance of the TMJ are a component of DJD. The American Academy of Orofacial Pain (AAOP) defines this disease entity as a type of abnormality characterized by erosion and progressive destruction of the articular surface of the condyle with associated remodeling of the underlying subchondral bone [63]. It is estimated to affect between 18% and 85% of patients clinically diagnosed with TMD and between 45% and 93% of those diagnosed with rheumatoid arthritis [9]. The final confirmation of degenerative joint disease, in addition to the clinical examination, is a radiological assessment which evaluates the presence of lesions such as osteophytes (local, marginal hypertrophic bony outgrowths on the condyle with sclerotic borders), subcortical and generalized sclerosis (increased density of cortical bone which extends into the bone marrow), subcortical cysts (rounded osteolytic lesions adjacent to subcortical bone area below the articular surface without cortical destruction), surface erosion (an area of decreased density resulting in loss of continuity of articular cortex), and articular surface flattening (a loss of the convex or concave contour of the articular surface).

The presence of joint space reduction and calcified loose bodies in soft tissues has also been examined. Pathologies in this area are not conclusive nor direct predictors of DJD even though are often present in its course [6].

The cause of degenerative changes is attributed to the remodeling of joint structures, when there is an imbalance between repair and destructive processes in cartilage and bone. This is a response to excessive or prolonged stress on the temporomandibular joints with a concomitant decrease in their adaptive capacity. Both a history of condylar trauma and increased joint friction (for example, due to degeneration of the synovial membrane) and disturbed, unstable occlusion contribute to the breakdown of compensatory mechanisms and initiation and lead to further changes. Vascular endothelial growth factor (VEGF), upregulating matrix metalloproteinases and their inhibitors (MMP-13 and TIMP-1), and proinflammatory cytokines (TNF-alpha, IL-1, and IL-6) are then stimulated and activated. This results in impaired synthesis and distribution of extracellular matrix and degradation of hyaluronic acid along with increased free radical activity in the TMJ, which is gradually covered by degenerative changes [64, 65]. Analyses of the results of studies conducted in recent years support this theory [11, 38, 39, 41].

Most researchers agree that severe skeletal and dental class II malocclusions, particularly those coexisting with a severely retrognathic mandible, as well as bony open bite patients (hypodivergent patients) characterized by a steep mandibular plane, are more likely to develop radiographically detectable degenerative changes [11, 38, 39, 42]. This correlation is also observed in patients with skeletal class III malocclusions exhibiting a mandibular shift (chin deviation > 3 mm) [41]. This view is consistent with most previous studies that evaluated degenerative changes in the temporomandibular joint using conventional tomography or pantomographic radiographs [36, 66–68]. Only one of the papers included in the systematic review showed no association of intra-articular pathologies with skeletal defects [40].

### 4.2. Joint Space and Condylar Position in the TMJ in CBCT: Summary

The position of the condyles in the TMJ may influence the development of dysfunction which includes osteoarthritis. A TMJ which compresses the retrodiscal tissues and coexists with severe muscle tension of the masticatory system is one of the main causes of the onset of pain [65]. Most of the conclusions from the included studies agree that skeletal class II malocclusion and patients in the skeletal open bite group with retrognathic mandibles have condylar processes positioned more anteriorly and superiorly in the articular joint space compared to the general population [34]. Patients in the skeletal deep bite group usually have narrowing of the posterior or superior articular joint spaces [22, 24, 54–56]. In class III defects, the head of the condylar process is usually positioned closer to the upper wall of the articular fossa [34, 35, 46, 57]. As with degenerative lesions, the presence of a lateral displacement of the chin to a significant extent (greater than 3 mm) can affect the malposition of the condylar heads in the joint and therefore be a risk factor for temporomandibular joint dysfunction [41].

### 4.3. TMJ Condylar Shape in CBCT: Summary

The shape of the articular eminence also influences the correct position of the condylar process, which among other things, affects the stability of the occlusion and the results of orthodontic treatment [30, 31]. The anatomy of the condyles and glenoid fossa, as well as the position of the condyle, may play an important predictive role in the accurate clinical identification of degenerative changes in the TMJ [15]. This is often the basis for developing temporomandibular joint dysfunction. The current prevalence of TMD among dental patients is a reason for the need to broaden the knowledge of dysfunction and the impact of maxillofacial morphology and occlusion due to the varying opinions of researchers [28, 69, 70]. The reviewed works show a flat or slightly convex head of the condylar process is the most common presentation in the general population [44, 45]. According to some researchers, patients in a class II malocclusion group are characterized by a significantly convex shape of the condylar process [45], while those in a high-angle group are oval or concave [24]. Kurusu et al. [20] demonstrated that the structure of the condylar process is related to the muscular forces affecting it. They showed that larger dimensions of the condyle are related to its loading. Furthermore, they demonstrated an increase in the forces applied to the head of the condylar process in patients in the brachyfacial group compared to the dolichofacial group. Taking into account the above findings and the position of the condylar process in the joint as well as depending on the malocclusion, it can be assumed that patients with bony deep occlusions will be significantly more likely to develop TMD compared to the rest of the population.

### 4.4. TMJ Articular Eminence and Mandibular Fossa in CBCT: Summary

Another important component of the TMJ whose abnormal anatomy may contribute to pathology is the glenoid fossa and articular eminence. Patients who have an angle that is lower or higher than normal in the general population (30° to 60°) may exhibit internal articulation disorders [71]. A low articular eminence may facilitate the articular disc to pass above the highest point during mandibular movements which significantly increases the probability of disc dislocation [72]. In the articles reviewed, it was most often shown that patients in a skeletal-dental class III malocclusion group were characterized by the relatively shallowest and widest articular fossa and, at the same time, the lowest and least steep articular eminence [34, 35, 46, 57]. These results suggest that patients in this group are significantly more likely to have pathologies in terms of articular disc displacement.

Clinicians' knowledge of potential risk factors for the development of temporomandibular joint dysfunction and degenerative changes should be continuously expanded and updated. Further research is necessary to clarify the relationship between the position of the condylar processes and the presence of degenerative changes in the temporomandibular joints in orthodontic patients. It may be helpful to recommend CBCT for the group of malocclusions in which degenerative changes occur most frequently. This would improve the diagnostic process and minimize errors in treatment planning.

## 5. Conclusions

Within the limitations of this review, the following conclusions may be drawn:

1. Dental and skeletal class II malocclusions with a retrognathic mandible as well as hypodivergent facial patterns with a steep mandibular plane may be risk factors for developing radiographically detectable degenerative changes in the TMJ; condyles are mostly positioned more anteriorly and superiorly in the articular fossa compared with the general population
2. Dental and skeletal class III malocclusion patients may be at greater risk of TMJ articular disc dislocation due to the anatomy of the articular fossa and eminence; condyles are commonly positioned more anteriorly in the articular fossa compared with the general population
3. Hyperdivergent patients have a higher risk of developing TMD in comparison with other groups
4. Correlation of the shape of the condyle with different dentofacial morphologies remains unclear

## Figures and Tables

**Table 1:** The inclusion and inclusion and exclusion criteria.

Inclusion criteria	Papers that evaluated the effects of orthodontic variables on the temporomandibular joint using cone-beam computed tomography imaging: (i) Malocclusion (ii) Occlusal disorders (iii) Cephalometric variables
	Papers that evaluated the effects of orthodontic variables on the temporomandibular joint using cone-beam computed tomography imaging of the temporomandibular joint: (i) Shape and volume of the condyle (ii) Measurements of the joint space (iii) Shape and size of the articular eminence and glenoid fossa (iv) Position of the condyles (v) Evaluation of degenerative changes in the temporomandibular joint
Exclusion criteria	(i) Case reports, comments, interviews, authors' debates, editorials, letters, and review articles (ii) Animal studies (iii) Articles published before 2009 (iv) Articles published in languages other than English (v) Assessments conducted with magnetic resonance imaging (MRI) or standard computed tomography (CT) (vi) In vitro studies (vii) Patients who underwent orthodontic treatment or splint therapy (viii) Less than 10 patients in the research group

**Table 2:** Summary of the descriptive characteristics of included articles regarding degenerative changes; ^∗^statistically significant; F: female; M: male; n/a: not available; kV: kilo voltage; mA: milliampere; FOV: field of view.

Reference	Population (country)	Average age (y.o.)	Gender	CBCT acquisitions	Number of patients/joints evaluated	Number of investigators evaluating changes in CBCT	Main group classification	Assessed orthodontic variables	Assessed degenerations	% of overall joints with TMJ osseous alterations	Most frequently occurring degenerations	Main correlations and conclusions
Kang et al. [39]	South Korea	14.2 (adolescents)	95 F, 0 M	100 kV, 9.0 mA, FOV 14 × 12 cm, 0.3 mm voxel	95/190	n/a	(1) By age and gender (2) By vertical facial pattern (3) By RDC/TMD classification	SNA, SNB, ANB, N-A, N-ANS, N-B, N-Pog, Ar-Go, S-Go/N-Me, N-S-Ar, Ar-Go-Me, N-Go-Ar, N-Go-Me, N-S-Gn, SN/Go-Me, FMA, FMIA, IMPA,	Flattening, osteophyte, erosion, sclerosis, resorption,	46.3%	n/a	(1) ^∗^More retrognathic mandible, larger FMA, *y*-axis to SN angle, SN-GoMe angle and lower facial height ratio (hyperdivergent pattern with steeper mandibular plane) in OA group (2) Retarded dental development in OA group
Dadgar-Yeganeh et al. [42]	USA	n/a (adolescents and adults)	198 F, 75 M	120 kV, 18.45-47.74 mA, FOV n/a, voxel n/a	273/546	4	(1) By vertical facial pattern	MP-SN	Remodeling, erosion, flattening, sclerosis, osteophytes, subcondylar bone cysts, joint space narrowing joint congruence	12.8%	n/a	(1) ^∗^Smaller condylar, ramus and mandibular height in DJD group (2) ^∗^Increased probability of having DJD in long vertical facial dimensions group (3) ^∗^smaller upper airway dimension in the DJD group patients
Chen et al. [38]	China	24.1 (adults)	83 F, 0 M	90 kV, 7.0 mA, FOV 16 × 10 cm, voxel n/a	83/166	1	(1) By skeletal sagittal pattern (2) By vertical facial pattern (3) By condylar features	SNA, SNB, ANB, MP-SN, Pg to *y*-axis, A to *y*-axis, S-Go, N-Me, S-Go/N-Me(%)	Flattening, osteophyte, erosion, subcortical sclerosis, subcortical cyst, hypoplasia,short condyle, generalized sclerosis	52.4%	Short condyles	(1) ^∗^The smallest S-Go, the highest MP-SN and the most retruded mandible in the OA group
Walewski et al. [40]	Brazil	n/a (adults)	116 F, 97 M	120 kV, 38 mA, FOV n/a, 0.3 mm voxel	213/426	2	(1) By age (2) By skeletal sagittal pattern	ANB angle (skeletal classes I, II, and III)	Flattening, osteophyte, erosion, deviation in form, subcortical sclerosis, subcortical cyst, hypoplasia	52.3%	Flattening	(1) No differences regarding OA among different skeletal patterns, gender or age groups
Krisjane et al. [11]	Latvia	20.3 (adolescents and adults)	n/a	120 kV, 38 mA, FOV 17 × 17 cm, 0.4 mm voxel	117/234	1	(1) By skeletal sagittal pattern	ANB angle (skeletal classes I, II, and III)	Flattening, osteophyte, erosion, deviation in form, subcortical sclerosis, subcortical cyst, hypoplasia, hyperplasia	42.7%	Flattening	(1) ^∗^The highest OA incidence in skeletal and dental class II malocclusion (2) ^∗^Higher incidence of degenerative changes in skeletal class II and III malocclusion compared with class I
Tran Duy et al. [41]	Taiwan	24.5 (adults)	85 F, 0 M	120 kV, 38 mA, FOV 16 × 22 cm, 0.4 mm voxel	85/170	2	(1) By skeletal sagittal pattern (2) By chin deviation	SNA, SNB, FMA, SN-GoGn, Wits, Pog-Nv, overbite, overjet	Flattening, osteophyte, erosion, subcortical sclerosis, pseudocyst, generalized sclerosis	n/a	n/a	(1) ^∗^Higher incidence of degenerative changes in skeletal class III malocclusion patients with ≥3 mm chin deviation (2) ^∗^correlation of the joint volume differences and subcortical sclerosis with chin deviation

**Table 3:** Summary of the descriptive characteristics of included articles regarding TMJ condylar shape; ^∗^statistically significant; F: female; M: male; n/a: not available; kV: kilo voltage; mA: milliampere; FOV: field of view.

Reference	Population (country)	Average age (y.o.)	Gender	CBCT acquisitions	Number of patients/joints evaluated	Number of investigators evaluating changes in CBCT	Main group classification	Assessed orthodontic variables	CBCT plane for the condyle assessment	Classification by the condyle shape	% distribution of condyles according to the assigned classification	Main correlations and conclusions
Yalcin et al. [45]	Turkey	42.31 (adults)	458 F, 452 M	120 kV, 5 mA, FOV 16 × 9 and 16 × 16 cm	910/1820	2	(1) By Angle's classification	Class I, II, and III molar relationship	Coronal	(1) Convex (2) Round (3) Flat (4) Angled	(1) Convex (40.6%) (2) Angled (34.3%) (3) Flat (15.4%) (4) Round (9.7%)	(1) ^∗^Correlation between Angle's classification and shape of the condyle in the right condyle, no correlation in the left condyle (2) Mostly convex shape of the condyle in Angle's class II malocclusion patients
Merigue et al. [44]	Brazil	16.4 (adolescent and adults)	F and M (detailed distribution n/a)	120 kV, 36.9 mA, FOV 13 × 23 cm	49/98	1	(1) By Angle's classification	Class I and II division 1 molar relationship	Coronal	(1) Round (2) Flat or convex (3) Triangular or angled	(1) Flat or convex (66%) (2) Round (28%) (3) Triangular or angled (6%)	(1) No correlation between malocclusion and shape of the condyle (2) Convex shape was the most prevalent in all groups
Kurusu et al. [20]	Japan	24 (adults)	40 F	80 kV, 7 mA, FOV n/a	40/80	1	(1) By sagittal skeletal pattern and Angle's classification	SNA, SNB, Occ-FH, MP-FH, Gonial angle, ramus inclination N-Me, S-Go	Coronal and axial	Individual assessment at 30° intervals form the center of the long axis	n/a	(1) ^∗^Correlation between occlusal forces and mandibular plane angle-the bigger MP-FH the lower the forces (2) ^∗^Smaller condyles of low-occlusal-force patients than high-occlusal-force patients
Park et al. [24]	South Korea	25.52 (adults)	34 F, 26 M	80 kV, 5 mA, FOV 20 × 17.9 cm	60/120	1	(1) By vertical facial pattern	SN-GoMe angle	Saggital	1. Normal (round and oval) 2. Flattened 3. Osteophytic 4. Unclassified	(1) Normal (72.5%) (2) Flattened (20%) (3) Osteophytic (7.5%) (4) Unclassified (0%)	(1) ^∗^Hypodivergent group showed significant ratio of oval condyles (2) ^∗^Hyperdivergent group showed significant ratio of round condyles (3) ^∗^Condyles in hyperdivergent group have smaller dimensions than the hypodivergent ones
Contro et al. [43]	USA	n/a (adults)	169 F, 73 M	120 kV, 18.45-47.74 mA, FOV n/a	242/484	1	(1) By vertical facial pattern (2) By mandibular symphysis morphology	MP-SN, Ar-Go-Me, S-Go/N-Me, Id-Pg-MP (mandibular symphysis morphology)	Coronal, axial and saggital	Individual assessment based on Procrustes analysis	n/a	(1) Axial view: dolichofacial group showed concave anterior surface of the condyle, brachyfacial group showed convex anterior surface (2) Averages in acute chin angle group were similar to the dolichofacial group (3) Averages in obtuse chin angle group were similar to the brachyfacial group

**Table 4:** Summary of the descriptive characteristics of included articles regarding joint space and condylar position; ^∗^statistically significant; F: female; M: male; n/a: not available; kV: kilo voltage; mA: milliampere; FOV: field of view; TMDs: temporomandibular joint disorders.

Reference	Population (country)	Average age (years)	Gender	CBCT acquisitions	Number of patients/joints evaluated	Number of investigators evaluating changes in CBCT	Main group classification	Assessed orthodontic variables	CBCT plane for joint space assessment	Mutual relationship of dental arches during CBCT acquisition	Main correlations and conclusions
Lin et al. [22]	China	22.46 (adolescents and adults)	60 F, 0 M	120 kV, 5 mA, FOV 17 × 23 cm	60/120	1	(1) By vertical and dentoskeletal facial pattern	FH-MP, SN-GoGN, skeletal pattern, molar class relationship	Sagittal	Maximal intercuspal position	(1) ^∗^Correlation regarding posterior joint space: highest values in the high-angle group and lowest in the low-angle group (2) ^∗^Correlation regarding superior joint space: substantially lower values in the high-angle group (3) ^∗^Anterior joint space-substantially higher values in the low-angle group (4) ^∗^Anterior and concentric positions in the high-angle group; concentric positions in the control group; posterior and concentric positions in low-angle groups (5) Higher risk of TMD in skeletal class II malocclusion with a high angle
Noh et al. [55]	South Korea	23.5 (adults)	83 F, 48 M	80 kV, 5.0-8.0 mA, FOV 15.4 × 15.4 cm	131/262	1	(1) By skeletal sagittal pattern (2) By vertical facial pattern	SN-MP, ANB. Angle (skeletal classes I, II, and III)	Sagittal	n/a	(1) ^∗^Superior joint space: lower values in the hyperdivergent patient group compared with other groups (2) No differences in joint space between sagittal skeletal patterns
Arieta-Miranda et al. [34]	Peru	25 (adults)	n/a	90 kV, 8 mA, FOV 20 × 19 cm	45/90	2	(1) By skeletal sagittal pattern (2) By vertical facial pattern	ANB angle (skeletal classes I, II, and III), SGo/Nme relationship	Sagittal	Maximal intercuspal position	(1) ^∗^Superior joint space: lower values in skeletal the class II and III group compared to the class I group (2) ^∗^Anterior joint space-higher values in skeletal class I compared to the class II and III group (3) No differences in posterior joint space
Chae et al. [54]	South Korea	n/a (adolescents)	64 F, 54 M	80 kV, 60 mA, FOV 19.97 × 19.97 cm	120/240	1	(1) By skeletal sagittal pattern (2) By vertical facial pattern	ANB angle (skeletal classes I, II, and III), Sgo/Nme relationship	Sagittal and frontal	Centric occlusion	(1) No differences in condylar position in the glenoid fossa (2) Hypodivergent group: condyles were seated more inferiorly in the glenoid fossa in comparison with the hyperdivergent class II group
Paknahad et al. [25]	Iran	25.2 (adults)	n/a	120 kV, 4.6 mA, FOV 15 × 15 cm	60/120	1	(1) By skeletal sagittal pattern	ANB angle (skeletal classes I, II, and III)	Sagittal	Maximal intercuspal position	(1) ^∗^Anterior joint space: lower values in class II compared to classes I and III (2) No differences in condylar position between class I and III groups (3) The most frequent position: class I concentric (53.3%); class II anterior (50%); class III concentric (46.7%)
Song et al. [46]	China	18.11 (adolescents and adults)	63 F, 60 M	n/a	123/246	1	(1) By skeletal sagittal pattern	ANB angle (skeletal classes I, II and III), FMA angle, APDI, position of the anterior teeth, overbite	Sagittal	n/a	(1) ^∗^Posterior joint space: higher values in the class II division 2 group compared with the other groups (2) Superior joint space: the smallest values in the class III group compared to classes I and II
Gorucu-Coskuner et al. [51]	Turkey	11.4 (adolescents)	14 F, 14 M	120 kV, 3.8 mA, FOV 19 × 24 cm	28/48	1	(1) By skeletal sagittal pattern	ANB angle (skeletal class II div 1 and 2),	Sagittal and axial	Maximal intercuspal position	(1) ^∗^Anterior joint space: higher values in class II division 1 group compared to the class II division 2 group (2) No differences in condylar position in other diameters
Kaur et al. [48]	India	n/a (adults)	n/a	85 kV, 10 mA, FOV 17 × 13.5 cm	45/90	1	(1) By skeletal sagittal pattern	ANB angle (skeletal classes I, II, and III)	Sagittal and frontal	Maximal intercuspal position	(1) Sagittal plane condyles position: class I: anterosuperior; class II: posterosuperior; class III: more anterosuperior than in class I (2) Frontal plane condyles position: mediolateral in all groups (3) ^∗^Axial plane: diameter of the condyle and angulation of the condylar process to midsagittal plane differs between all groups
Alhammadi et al. [56].	Egypt	n/a (adults)	n/a	n/a	60/120	2	(1) By skeletal sagittal pattern	ANB angle (skeletal classes I, II, and III)	Sagittal, axial, and frontal	n/a	(1) ^∗^Anterior joint space: highest values in the class II group (2) ^∗^Medial and superior joint space: lowest values in class III group (3) Skeletal class II malocclusion patients can be more prone to TMDs than skeletal class III malocclusion patients
Mishra et al. [50]	Nepal	10.8 (adolescents)	8 F, 12 M	n/a	20/40	1	(1) By skeletal sagittal pattern	ANB angle (skeletal classes I and III)	Sagittal, axial, and frontal	n/a	(1) No differences in condylar position in the glenoid fossa
Lobo et al. [35]	Brazil	n/a (adults)	90 F, 90 M	120 kV, 38 mA, FOV 17 × 23 cm	180/360	2	(1) By skeletal sagittal pattern (2) By age and gender	ANB angle (skeletal classes I, II, and III)	Sagittal	Maximal intercuspal position	(1) No differences in anterior joint space (2) ^∗^Correlation regarding superior and posterior joint space: substantially lower values in class III malocclusion
Kim et al. [53]	South Korea	22.6	64 F, 56 M	120 kV, 47.7 mA, FOV 20 × 40 cm	120/240	1	(1) By skeletal sagittal pattern and chin deviation	ANB angle (skeletal classes I and III), chin deviation	Sagittal	n/a	(1) No statistically significant differences regarding anterior and posterior joint space (2) ^∗^Superior joint space: the lowest values in class III with deviation >3 mm
Akbulut et al. [52]	Turkey	22.07 (adults)	36 F, 24 M	120 kV, 20.27 mA, FOV 16 × 6 cm	60/120	1	(1) By Angle's classification	Class I, II, and III molar relationship	Panoramic reconstruction	Centric occlusion in maximal intercuspal position	(1) No differences in condylar position in the glenoid fossa
Stasiuk et al. [58]	Ukraine	n/a (adolescents and adults)	36 F, 34 M	n/a	70/140	n/a	(1) By Angle's classification	Class I, II, and III molar relationship	Midsagittal	n/a	(1) Optimal condylar position in the glenoid fossa (the 4/7 Gelb's position) was found in 11.43% of cases; regarding Angle's classification: class I group: 17.65%, class II group: 6.67%, class III group: 0%
Henriques et al. [47]	Brazil	n/a (adults)	n/a	n/a	20/40	1	(1) By Angle's classification	Class I, II, and III molar relationship	Sagittal and frontal	Centric relation and maximal intercuspal position	(1) No differences regarding centric relation and maximal intercuspal position among all of the groups (2) High diversity of condylar position among both centric occlusion and maximal intercuspal position in all of the groups
Merigue et al. [44]	Brazil	16.4 (adolescent and adults)	F and M (detailed distribution n/a)	120 kV, 36.9 mA, FOV 13 × 23 cm	49/98	1	(1) By Angle's classification	Class I and II division 1 molar relationship	Sagittal	n/a	(1) No statistically significant difference in condylar position in the glenoid fossa (2) In both of the groups more anteriorly condylar position were found in terms of concentricity in the glenoid fossa
Park et al. [24]	South Korea	25.52 (adults)	34 F, 26 M	80 kV, 5 mA, FOV 20 × 17.9 cm	60/120	1	(1) By vertical facial pattern	SN-GoMe angle	Sagittal	Centric occlusion in maximal intercuspal position	(1) ^∗^Superior joint space—the highest values in hypodivergent group, the lowest values in hyperdivergent group
Ganugapanta et al. [49]	India	n/a (adults)	n/a	60 kV, 30 mA, FOV n/a	60/120	n/a	(1) By vertical facial pattern	(vertical facial pattern values n/a), overbite	Sagittal and axial	Maximal intercuspal position	(1) No differences in condylar position between the control group and horizontal growth pattern with the deep bite group (2) ^∗^Posterior joint space: lower values in vertical growth pattern with the deep bite group compared with the control group (3) Facial growth pattern may have impact on the position of the condyles
Alhammadi et al. [56].	Egypt	n/a (adults)	n/a	n/a	60/120	2	(1) By vertical facial pattern	Mandibular and maxillary plane angle, *y*-axis angle, facial height ratio	Sagittal, axial, and frontal	n/a	(1) ^∗^Condylar position in long face group: higher values of posterior joint space, the highest medial joint space, lower vertical point and geometric condylar position, more lateral positioned condyles compared to average and short face groups (2) ^∗^Condylar position in short face group: higher values of vertical point and geometric condylar position, the highest superior and anterior and the lowest posterior joint spaces (3) Short face malocclusion patients can be more prone to TMDs than long face or average face patients

**Table 5:** Summary of the descriptive characteristics of included articles regarding TMJ articular fossa/eminence; ^∗^statistically significant; F: female; M: male; n/a: not available; kV: kilo voltage; mA: milliampere; FOV: field of view.

Reference	Population (country)	Average age (years)	Gender	CBCT acquisitions	Number of patients/joints evaluated	Number of investigators evaluating changes in CBCT	Main group classification	Orthodontic variables assessed	Evaluated articular eminence and glenoid fossa features	Main correlations and conclusions
Lin et al. [22]	China	22.46 (adolescents and adults)	60 F	120 kV, 5 mA, FOV 17 × 23 cm	60/120	1	(1) By vertical facial pattern (2) By skeletal sagittal pattern (3) By Angle's classification	FH-MP angle, SN-GoGN angle, skeletal pattern, molar class relationship	(1) Depth and width of the glenoid fossa (2) Inclination and height of the articular eminence	(1) No differences in width of the glenoid fossa and height of the articular eminence (2) ^∗^Depth of the glenoid fossa: smaller values in the high-angle group (3) Inclination of the articular eminence: the highest values in the low-angle group and the lowest in the high-angle group (4) Skeletal class II malocclusion with high angle may be associated with unstable TMJ
Arieta-Miranda et al. [34]	Peru	25 (adults)	n/a	90 kV, 8 mA, FOV 20 × 19 cm	45/90	2	(1) By vertical facial pattern (2) By skeletal sagittal pattern	ANB angle (skeletal classes I, II, and III), SGo/NMe relationship	(1) Inclination and height of the articular eminence	(1) Articular eminence inclination: the skeletal class III group showed smaller values than the skeletal class II group (2) Both classes II and III showed smaller inclination than the class I group (3) ^∗^height of the articular eminence: smaller values in class III group than in class I group
Noh et al. [55]	South Korea	23.5 (adults)	83 F, 48 M	80 kV, 5.0-8.0 mA, FOV 15.4 × 15.4 cm	131/262	1	(1) By skeletal sagittal pattern (2) By vertical facial pattern	SN-MP, ANB. Angle (skeletal classes I, II, and III)	(1) Length and height of the glenoid fossa	(1) ^∗^The skeletal class III group showed larger values of glenoid fossa height compared with skeletal class II (2) ^∗^The hyperdivergent group showed larger values of glenoid fossa length and height compared with other groups
Chae et al. [54]	South Korea	n/a (adolescents)	64 F, 54 M	80 kV, 60 mA, FOV 19.97 × 19.97 cm	120/240	1	(1) By vertical facial pattern (2) By skeletal sagittal pattern	ANB angle (skeletal classes I, II, and III), SGo/NMe relationship	(1) Inclination of the articular eminence (2) Depth of the glenoid fossa	(1) No differences in inclination of the articular eminence and depth of the glenoid fossa
Walewski et al. [40]	Brazil	n/a (adults)	116 F, 97 M	120 kV, 38 mA, FOV n/a	213/426	2	(1) By skeletal sagittal pattern (2) By age	ANB angle (skeletal classes I, II, and III)	(1) The presence or absence of articular fossa/eminence surface flattening, erosion, and subcortical sclerosis	(1) In total, 31.5% articular fossae/eminences showed degenerative alterations (2) Flattening most prevalent lesion (3) No differences regarding degenerative alterations of articular fossae/eminences
Lobo et al. [35]	Brazil	n/a (adults)	90 F, 90 M	120 kV, 38 mA, FOV 17 × 23 cm	180/360	2	(1) By skeletal sagittal pattern (2) By age and gender	ANB angle (skeletal classes I, II, and III)	(1) Inclination and height of the articular eminence (2) Thickness of the roof of the glenoid fossa	(1) ^∗^Inclination and height of the articular eminence: smaller values in the skeletal class III and I group (2) ^∗^Thickness of the roof of the glenoid fossa: the lowest values in the skeletal class III and I group (3) No differences between right and left side of the patient
Song et al. [46]	China	18.11 (adolescents and adults)	63 F, 60 M	n/a	123/246	1	(1) By skeletal sagittal pattern	ANB angle (skeletal classes I, II, and III), FMA angle, APDI, position of the anterior teeth, overbite	(1) Depth and width of the glenoid fossa	(1) ^∗^Wider glenoid fossa in the skeletal class III group compared to the other groups (2) ^∗^Smaller depth of the joint fossa in the class III group compared the other groups
Moscagiuri et al. [60]	Italy	25 (adults)	28 F, 24 M	80-110 kV, 5.0-9.0 mA, FOV 19 × 2 cm	52/104	2	(1) By skeletal sagittal pattern	ANB angle (skeletal classes I, II, and III), WITS, A-Pg,	(1) Inclination of the articular eminence	(1) No differences between right and left side of the patient (2) No differences between divergent skeletal sagittal pattern
Fan et al. [59]	China	27.91 (adults)	41 F, 26 M	110 kV, 5.0 mA, FOV 18 × 16 cm	67/134	2	(1) By skeletal sagittal pattern	ANB angle (skeletal class I), molar class relationship (I, II/1, II/2), FH-MP, SN-GnGo (normodivergent)	Height and inclination of the articular eminence Depth and width of the glenoid fossa	(1) ^∗^The highest inclination of articular eminence in the class II/2 patient group (2) ^∗^Difference between class I and class II patients groups regarding inclination of the articular eminence (3) ^∗^The widest glenoid fossa in the class I patient group (4) No differences in depth of the glenoid fossa and height of the articular eminence
Khademi et al. [61]	Iran	n/a (adults)	n/a	n/a	64/128	n/a	(1) By skeletal sagittal pattern	ANB angle (skeletal classes I, II, and III)	(1) Angle, depth, and width of the glenoid fossa	(1) ^∗^Wider glenoid fossa in the skeletal class I group compared with the skeletal class III group (2) No differences in angle and depth of the glenoid fossa
Gorucu-Coskuner et al. [51]	Turkey	11.4 (adolescents)	14 F, 14 M	120 kV, 3.8 mA, FOV 19 × 24 cm	28/48	1	(1) By skeletal sagittal pattern	ANB angle (skeletal class II div 1 and 2,	(1) Depth and width of the glenoid fossa	(1) ^∗^Deeper and wider glenoid fossa in the class II division 1 group compared to the class II division 2 group
Alhammadi et al. [56].	Egypt	n/a (adults)	n/a	n/a	60/120	2	(1) By skeletal sagittal pattern	ANB angle (skeletal classes I, II, and III)	(1) Height of the articular eminence, depth, width, and other linear and angular measurements of the glenoid fossa	(1) ^∗^The highest vertical inclination in the class II group compared to the other groups (2) ^∗^More inferior position, the highest width and posterior wall inclination of the glenoid fossa in the class III group compared to the other groups (3) ^∗^Lower height of the articular eminence in class III group compared to the other groups
Park et al. [24]	South Korea	25,52 (adults)	34 F, 26 M	80 kV, 5 mA, FOV 20 × 17.9 cm	60/120	1	(1) By vertical facial pattern	SN-GoMe angle	(1) Inclination of the posterior wall of the articular eminence (2) Depth of the glenoid fossa	(1) No differences in inclination of the articular eminence (2) ^∗^Depth of the glenoid fossa (left condyles): the lowest values in the hyperdivergent group
Alhammadi et al. [56].	Egypt	n/a (adults)	n/a	n/a	60/120	2	(1) By vertical facial pattern	Mandibular and maxillary plane angle, *y*-axis angle, facial height ratio	(1) Height of the articular eminence, depth, width and other linear and angular measurements of the glenoid fossa	(1) ^∗^Highest glenoid fossa width and lowest anterior articular inclination in long face group compared to the other groups (2) ^∗^Increased anterior wall inclination, highest surface area, and anterior articular inclination of glenoid fossa in THE short face group compared to the other groups
Ganugapanta et al. [49]	India	n/a (adults)	n/a	60 kVp, 30 mA FOV n/a	60/120	n/a	(1) By vertical facial pattern	(vertical facial pattern values n/a), overbite	(1) Depth and width of the glenoid fossa	(1) No differences in depth of the glenoid fossa according to dental and vertical facial pattern

## Data Availability

The data used to support the findings of this study are included within the article.

## Supplementary Materials

Supplementary Materials Search Strategy example: PubMed in process and other nonindexed citations and Ovid MEDLINE, 1946–present.
