# Supplementary material for: Assessment of the Morphology and Degenerative Changes in the Temporomandibular Joint Using CBCT according to the Orthodontic Approach: A Scoping Review
Source: Biomed Res Int. 2022 Feb 1;2022:6863014. doi: 10.1155/2022/6863014 (PMC8826117; doi:10.1155/2022/6863014)
Supplement: Supplementary Materials — Search Strategy example: PubMed in process and other nonindexed citations and Ovid MEDLINE, 1946–present. [file 6863014.f1.zip › Appendix November 2021 revised.docx]

Search Strategy Example: PubMed in Process and Other Nonindexed Citations and Ovid MEDLINE, 1946–Present

**APPENDIX :**

| No. | Searches for section 2.2 | Results for  headwords only/full search |
| --- | --- | --- |
| 1 | „orthodontic” or „orthodontics” | 86 119 / 235 |
| 2 | „malocclusion" | 37 754 / 108 |
| 3 | „cephalometric" or „cephalometry" | 29 540 / 64 |
| 4 | "sagittal skeletal” or "sagittal relationship” or "skeletal relationship” | 411 / 10 |
| 5 | "skeletal pattern” or "anb angle” or "facial pattern” | 1346 / 14 |
| 6 | "occlusal relationship” | 370 / 2 |
| 7 | "dentofacial deformities” or "dentofacial deformity” | 1008 / 7 |
| 8 | "dentofacial orthop” | 9019 / 26 |
| 9 | "angle class” | 10 308 / 59 |
| 11 | 1 or 2 or 3 or 4 or 5 or 6 or 7 or 8 or 9 | 109 273 / 296 |
| **No.** | **Searches for section 2.3** | Results for  headwords only/full search |
| 1 | "osseous alterations” or "osseous abnormalities” or "osseous deformities” or "osseous components” or "bony changes” or "osseous changes” | 1655 / 47 |
| 2 | „osteoarthritis" or „osteoarthrosis" | 99 842 / 317 |
| 3 | "subcortical cysts” or "bone cysts” | 6385 / 6 |
| 4 | "surface erosion” or "bone erosion” or "surface flattening” | 2853 / 11 |
| 5 | "pathological findings” | 15 384 / 8 |
| 6 | "articular eminence” | 422 / 92 |
| 7 | "degenerative joint disease” | 2643 / 33 |
| 8 | "joint space” | 5585 / 124 |
| 9 | "condylar morphology” or "condylar head” | 645 / 142 |
| 10 | "skeletal structure” | 573 / 4 |
| 11 | „osteophyte” | 2798 / 28 |
| 12 | 1 or 2 or 3 or 4 or 5 or 6 or 7 or 8 or 9 or 10 or 11 | 132 613 / 688 |
